# Uncommon subtypes of frontal fibrosing alopecia: retrospective analysis of clinical characteristics and prognosis^☆^

**DOI:** 10.1016/j.abd.2021.02.009

**Published:** 2021-12-08

**Authors:** Vanessa Barreto Rocha, Carla Jorge Machado, Leticia Arsie Contin

**Affiliations:** a Hospital das Clínicas, Universidade Federal de Minas Gerais, Belo Horizonte, MG, Brazil; b Department of Public Health, Faculdade de Medicina, Universidade Federal de Minas Gerais, Belo Horizonte, MG, Brazil; c Dermatology Clinic, Hospital do Servidor Público Municipal de São Paulo, São Paulo, SP, Brazil

Dear Editor,

Frontal fibrosing alopecia (FFA) is a type of progressive lymphocytic scarring alopecia described by Kossard in 1994,^1^ which has become epidemic in recent years.^2^ It mainly affects the frontal and temporal areas of hair implantation. Three typical patterns have been described to date: linear (or type I), type II or diffuse (zigzag), and type III or pseudo fringe type. Each pattern has a different course, and the best prognosis is seen in the pseudo fringe pattern.^3^

The literature mentions some atypical FFA patterns: the plaque pattern,^4^ the male pattern, and the ophiasic pattern,^5^, ^6^ and their prognosis has not been described. The plaque pattern is characterized by areas of scarring alopecia that affect the temporal region, usually bilateral and symmetrical. During its course, there is loss of vellus hairs at the front edge leading to an irregular appearance of the hair implantation line.^4^ The male pattern is characterized by progressive alopecia, following a frontal recess pattern similar to that of male androgenetic alopecia.^5^ In the ophiasic pattern, the entire hair implantation line of the scalp is affected, the frontal region is affected in a straight or zigzag fashion (types previously classified as I and II), and the occipital region is also affected.^6^, ^7^

In a retrospective study of a series of 97 patients with FFA treated at the Clinic of Trichology of the Dermatology Service at HSPM-SP, from 2011 to 2019, 27 (27.8%) patients were identified with atypical patterns (Fig. 1): 12 (12.4%) with the male pattern, seven (7.2%) with the plaque pattern, and eight (8.2%) with the ophiasic pattern (Fig. 1). The study protocol was approved by the Research Ethics Committee of the HSPM-SP (N. 27274919.0.0000.5442).

**Figure 1:**
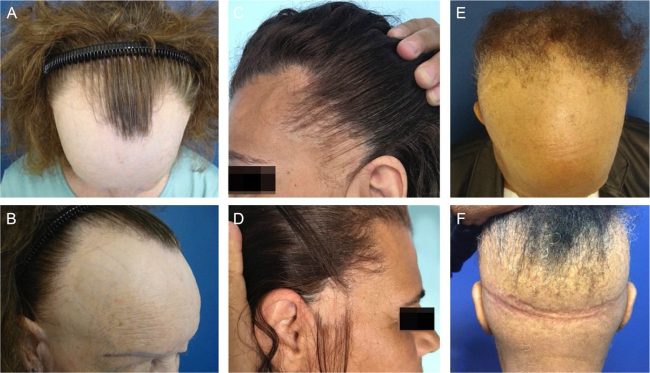
(A and B), Male pattern of FFA; (C and D), Plaque pattern; (E and F), Ophiasic pattern.

The patients were evaluated clinically and by photography, trichoscopy and measurement of the hair implantation line. The diagnostic criteria included progressive, symmetrical, and scarring alopecia with the absence of vellus hairs. A biopsy was performed to confirm the diagnosis. Disease stabilization was defined as no photographic progression of the alopecia, negative anagen traction test, absence of pruritus, pain, or burning sensation.

Table 1 summarizes the demographic data, clinical presentation, and comorbidities of 27 patients with uncommon FFA patterns. For the statistical analysis, the significance level was set at 5% (p < 0.05).

**Table 1:** Comparison of selected features of three unusual FFA subtypes.

Atypical FFA pattern	Male pattern	Plaque pattern	Ophiasic pattern	p value				Total
	n = 12 (100.0%)	n = 7 (100.0%)	n = 8 (100.0%)	Male × Plaque	Male × Ophiasic	Plaque × Ophiasic	Overall	n = 27 (100%)
Age: mean (SD)	65.3 (7.3)	60.7 (8.9)^a^	64.4 (14.4)	0.260	0.831	0.508	0.693	63.9 (10.0)^b^
Fitzpatrick II–III phototype^c^: n (%)	6 (50.0)	4 (57.1)	1 (12.5)	0.764	0.085	0.067	0.133	11 (40.7)
Approximate progression (cm/year): mean (SD)	1.02 (1.07)^d^	0.67 (0.51)^e^	2.25 (1.22)^f^	0.581	0.026^i^	0.038^i^	0.026^i^	1.31 (1.18)^g^
Stability: n (%)	8 (66.7)	4 (57.1)	1 (12.5)	0.678	0.017^i^	0.067	0.054	13 (48.1)
Disease duration until stabilization (months):mean (SD)	27.0 (16.0)^h^	18.8 (6.7)^h^	36 (0)^h^	0.503	–	–	0.474	25.2 (13.6)^h^
Disease evolution (years): mean (SD)	6.7 (5.3)	7.5 (6.4)^a^	6.7 (3.4)^a^	0.564	0.931	0.640	0.944	6.9 (5.0)
Facial papules: n (%)	4 (33.3)	0 (0.0)	2 (25.0)	0.086	0.690	0.155	0.284	6 (22.2)
Lichen planus pigmentosus: n (%)	3 (25.0)	0 (0.0)	3 (37.5)	0.149	0.550	0.070	0.240	6 (22.2)
Eyebrow alopecia: n (%)	11 (91.7)	0 (0.0)	7 (87.5)	<0.001^i^	0.761	<0.001^i^	<0.001^i^	18 (66.7)
Comorbidities: n (%)								
Systemic arterial hypertension	6 (50.0)	6 (85.7)	6 (75.0)	0.120	0.264	0.605	0.281	18 (66.7)
Diabetes mellitus	2 (16.7)	3 (42.9)	3 (37.5)	0.211	0.292	0.833	0.346	8 (29.6)
Dyslipidemia	4 (33.3)	2 (28.6)	2 (25.0)	0.830	0.690	0.876	0.935	8 (29.6)
Hypothyroidism	3 (25.0)	2 (28.6)	1 (12.5)	0.865	0.494	0.438	0.661	7 (25.9)

Notes: 2 × 2 comparisons of continuous variables were carried out using Student's *t* test for independent samples; 3 × 2 comparisons of continuous variables used the Analysis of Variance (ANOVA) table; percentage comparisons used Fisher's exact test; SD, Standard Deviation.

a n = 6.

b n = 26.

c in comparison with Fitzpatrick phototype IV–VI.

d n = 11.

e n = 5.

f n = 7.

g n = 15.

h only for patients who reported stability; Stata 12.0/SE for Mac was used.

i p < 0.05; the approximate progression for those without full follow-up was estimated as a direct proportion of the presumed one-year progression.

The patients included 26 women (24 post-menopausal), mean age 63.9 years (35–84 years, Standard Deviation – SD – 10). The phototypes ranged from II to VI on the Fitzpatrick scale (six II, six III, two IV, nine V, five VI), with no overall difference between the patterns (p = 0.133). The mean disease duration was 6.9 years. Six patients had facial papules (four with male pattern and two with ophiasic pattern), six patients had lichen planus pigmentosus (three with male pattern and three with ophiasic pattern, 83.3% of phototypes V and VI), and two patients had both lesions (one with male pattern and one with ophiasic pattern), with no statistical difference (p = 0.284 and 0.240 respectively).

Thirteen patients were considered stable; 14 had active disease, and two were lost to follow-up (excluded), with the ophiasic pattern being the least stable (p = 0.054).

The patients’ mean time of follow-up was 42.8 months (6–96 months, SD = 26.6).

When comparing disease progression, the ophiasic pattern showed the most aggressive progression (2.25 cm/year, SD = 1.22; p = 0.026) with no statistical difference between the male pattern (1.02 cm/year, SD = 1 .07) and the plaque pattern (0.67 cm/year, SD = 0.51; p = 0.581). Similar findings were observed regarding disease stability (p = 0.054 for greater progression of the ophiasic pattern).

Regarding the loss of eyebrows, the plaque pattern did not include patients with this type of alopecia (p < 0.001), and there was no statistical difference between the male and ophiasic patterns (p = 0.761), with 11 (91.7%) and seven patients (87.5%) with madarosis, respectively.

The main comorbidities observed were arterial hypertension, diabetes mellitus, dyslipidemia, and hypothyroidism, which were frequent, as expected, in this age group, with no statistical differences between the groups.

The demographic data, ethnicity, disease duration, and mean age at disease onset in our study were in line with other rare reports of atypical patterns of FFA.^4^, ^6^ However, the proportion of each atypical pattern varies widely between studies. Kanti et al., in a series of 490 patients with FFA, found 32% with the ophiasic pattern (called the “band-like” pattern).^8^ Rossi et al. described this pattern in 6.1% of their patients with FFA. These authors also found 12.2% of patients with the male pattern and 2.0% with the plaque pattern (called the “headdress” pattern).^6^ Recently, Goldman et al. described a case of fibrosing frontal alopecia with a symmetrical 'epsilon-shaped' alopecia pattern, which mimicked traction alopecia with symmetrical alopecia in the temporal area.^9^

In the present series, the plaque pattern, which would be the localized form of the disease, seems to have the best prognosis and the slowest evolution. These patients did not have manifestations such as facial papules and lichen planus pigmentosus, but there was no statistical difference from the other patterns.

The ophiasic pattern had a worse prognosis, and the male pattern had an intermediate prognosis. Both types showed the presence of facial papules, lichen planus pigmentosus and eyebrow alopecia, resembling cases of more aggressive disease.

The small number of patients, due to the lower incidence of atypical cases, is a limitation of the present study.

The study of these unusual presentations can help to improve the diagnosis and understand the prognosis of FFA.

## Financial support

None declared.

## Authors’ contributions

Vanessa B Rocha: Statistical analysis; approval of the final version of the manuscript; design and planning of the study; drafting and editing of the manuscript; collection, analysis, and interpretation of data; intellectual participation in propaedeutic and/or therapeutic conduct of the studied cases; critical review of the literature; critical review of the manuscript.

Carla Jorge Machado: Statistical analysis; approval of the final version of the manuscript; design and planning of the study; collection, analysis, and interpretation of data; effective participation in research orientation; critical review of the manuscript.

Leticia A. Contin: Approval of the final version of the manuscript; design and planning of the study; drafting and editing of the manuscript; collection, analysis, and interpretation of data; effective participation in research orientation; intellectual participation in propaedeutic and/or therapeutic conduct of the studied cases; critical review of the literature; critical review of the manuscript.

## Conflicts of interest

None declared.

## References

[1] Kossard S. (1994). Postmenopausal frontal fibrosing alopecia. Scarring alopecia in a pattern distribution. Arch Dermatol.

[2] Mirmirani P., Tosti A., Goldberg L., Whiting D., Sotoodian B. (2019). Frontal Fibrosing Alopecia: An Emerging Epidemic. Skin Appendage Disord.

[3] Moreno-Arrones O.M., Saceda-Corralo D., Fonda-Pascual P., Rodrigues-Barata A.R., Buendía-Castaño D., Alegre-Sánchez A. (2017). Frontal fibrosing alopecia: clinical and prognostic classification. J Eur Acad Dermatol Venereol.

[4] Contin L.A., Ledá Y.L.A., Caldeira Nassif K.C., Restrepo M.V.S. (2017). Patchy Frontal Fibrosing Alopecia: Description of an Incomplete Clinical Presentation. Skin Appendage Disord.

[5] Tremezaygues L., Vogt T., Muller C.S. (2012). [Frontal fibrosing alopecia with androgenetic pattern. A diagnostic challenge - a therapeutic problem].. Hautarzt..

[6] Rossi A., Grassi S., Fortuna M.C., Garelli V., Pranteda G., Caro G. (2017). Unusual patterns of presentation of frontal fibrosing alopecia: A clinical and trichoscopic analysis of 98 patients. J Am Acad Dermatol.

[7] Melo D.F., Barreto T.M., Faro G.B.A., Machado C.J., Donati A. (2020). Occipital hairline involvement in frontal fibrosing alopecia: frequency, clinical presentation and trichoscopy findings in a series of twenty patients. J Eur Acad Dermatol Venereol.

[8] Kanti V., Constantinou A., Reygagne P., Vogt A., Kottner J., Blume-Peytavi U. (2019). Frontal fibrosing alopecia: demographic and clinical characteristics of 490 cases. J Eur Acad Dermatol Venereol.

[9] Goldman C., Diaz A., Miteva M. (2020). A Novel Atypical Presentation of Frontal Fibrosing Alopecia Involving the Frontoparietal Scalp. Skin Appendage Disord.

